# Current News

**Published:** 1893-11

**Authors:** 


### Current News.

### MISSOURI STATE DENTAL ASSOCIATION.

The Twenty-ninth Annual Meeting of the Missouri State Dental Association was held at Excelsior Springs, Mo., July 11, 12, 13, 14. The following officers were elected for the ensuing year:

President, Dr. W. E. Tucker, Springfield; First Vice-President, Dr. J. T. Fry, Moberly; Second Vice-President, Dr. Charles L. Hungerford, Kansas City; Corresponding Secretary, Dr. William Conrad, St. Louis; Recording Secretary, Dr. S. C. A. Rubey, Clinton; Treasurer, Dr. James A. Price, Weston.

*Committee on Ethics.*—Dr. Frank Slater, Rich Hill; Dr. W. N. Morrison, St. Louis; Dr. C. L. Hungerford, Kansas City.

*Board of Censors.*—Dr. E. E. Shattuck, Kansas City; Dr. H. A. Cress, Warrensburg; Dr. E. B. Crane, California.

*Committee on Law.*—Dr. James A. Price, Weston.

*Committee on New Appliances.*—Dr. J. R. Harper, St. Louis.

The next meeting of this Association will be held at Excelsior Springs, Mo., on the first Tuesday after July 4, 1894.

William Conrad,
*Corresponding Secretary.*

St. Louis, Mo.

### RECENT PATENTS.

Following is a list of recent patents, reported specially for the International Dental Journal:

493,846. Dental Boring Apparatus. James Weber and Hugo Hempel, Berlin, Germany, assignors of one-half to Joseph Davidsohn, same place. Filed January 4, 1892. Patented in Germany, March 4, 1891.

493,893.—Dental Disk-Holder. Newton Morgan, Springfield, Mass. Filed December 19, 1892.

494,065.—Artificial Tooth-Plate. Gustav A. Juterbock, Berlin, Germany, assignor to Carl Otto Juterbock, Penge, England. Filed December 21, 1892.

497,122.—Dental Articulator. Charles F. Garretson, Knoxville, Iowa. Filed January 26, 1893.

497,370.—Brake for Dental Engines. Howard T. Eachus, St. Paul, Minn. Filed August 26, 1892.

497,723.—Dental Flask. Edwin A. Levering, Philadelphia, Pa. Filed March 6, 1893.

497,964.—Dental Hot-Air Syringe. Frank B. Norris, Helena, Montana. Filed November 8, 1892.

498,554.—Dental Broach. Olof Johanson, New York, N. Y. Filed January 21, 1893.

499,015.—Dental Furnace. James H. Downie, Detroit, Mich., assignor to the Detroit Dental Manufacturing Company, same place. Filed November 14, 1892.

499,550.—Rubber-Dam Clamp. James W. Ivory, Philadelphia, Pa. Filed March 5, 1892.

499,602.—Dental Vulcanizer. George R. Snow, Buffalo, N. Y. Filed July 8, 1891.

499,612.—Tooth-Brush. Daniel W. Tower, Grand Rapids, Mich. Filed December 21, 1892.

499,632.—Pneumatic Dental Plugger. John H. Heivly, Oil City, Pa. Filed October 3, 1892.

500,103.—Dental Chair. Daniel A. Nash, Jackson, Miss. Filed October 18, 1892.

---

### THE WOMAN'S DENTAL ASSOCIATION OF THE UNITED STATES.

The regular monthly meeting of the Woman's Dental Association was held October 7, 1893, at 1300 Arch Street, Philadelphia, President Mary H. Stilwell in the chair.

Professor C. N. Peirce read a paper entitled "Diagnosis."

The next meeting will be held at the same place November 4, 1893, at 7.30 P.M.

Eliza Yerkes,
*Recording Secretary.*

4004 Chestnut Street, Philadelphia.

